# Supplementary material for: Halorotetin A: A Novel Terpenoid Compound Isolated from Ascidian Halocynthia rotetzi Exhibits the Inhibition Activity on Tumor Cell Proliferation
Source: Mar Drugs. 2023 Jan 12;21(1):51. doi: 10.3390/md21010051 (PMC9860651; doi:10.3390/md21010051)
Supplement: Supplementary file 1 [file marinedrugs-21-00051-s001.zip › marinedrugs-2127434-supplementary.pdf]

Supplementary materials

Halorotetin A: A Novel Terpenoid Compound Isolated from Ascidian *Halocynthia rotetzi* Exhibits the Inhibition Activity on Tumor Cell Proliferation

Jianhui Li [1,†], Shanhao Han [1,†], Yuting Zhu [1] and Bo Dong [1,2,3,*]

[1] Fang Zongxi Center, MoE Key Laboratory of Marine Genetics and Breeding, College of Marine Life Sciences, Ocean University of China, Qingdao 266003, China;
[2] Laoshan Laboratory, Qingdao 266237, China
[3] Institute of Evolution & Marine Biodiversity, Ocean University of China, Qingdao 266003, China
* Correspondence
† These authors contribute equally to this work.

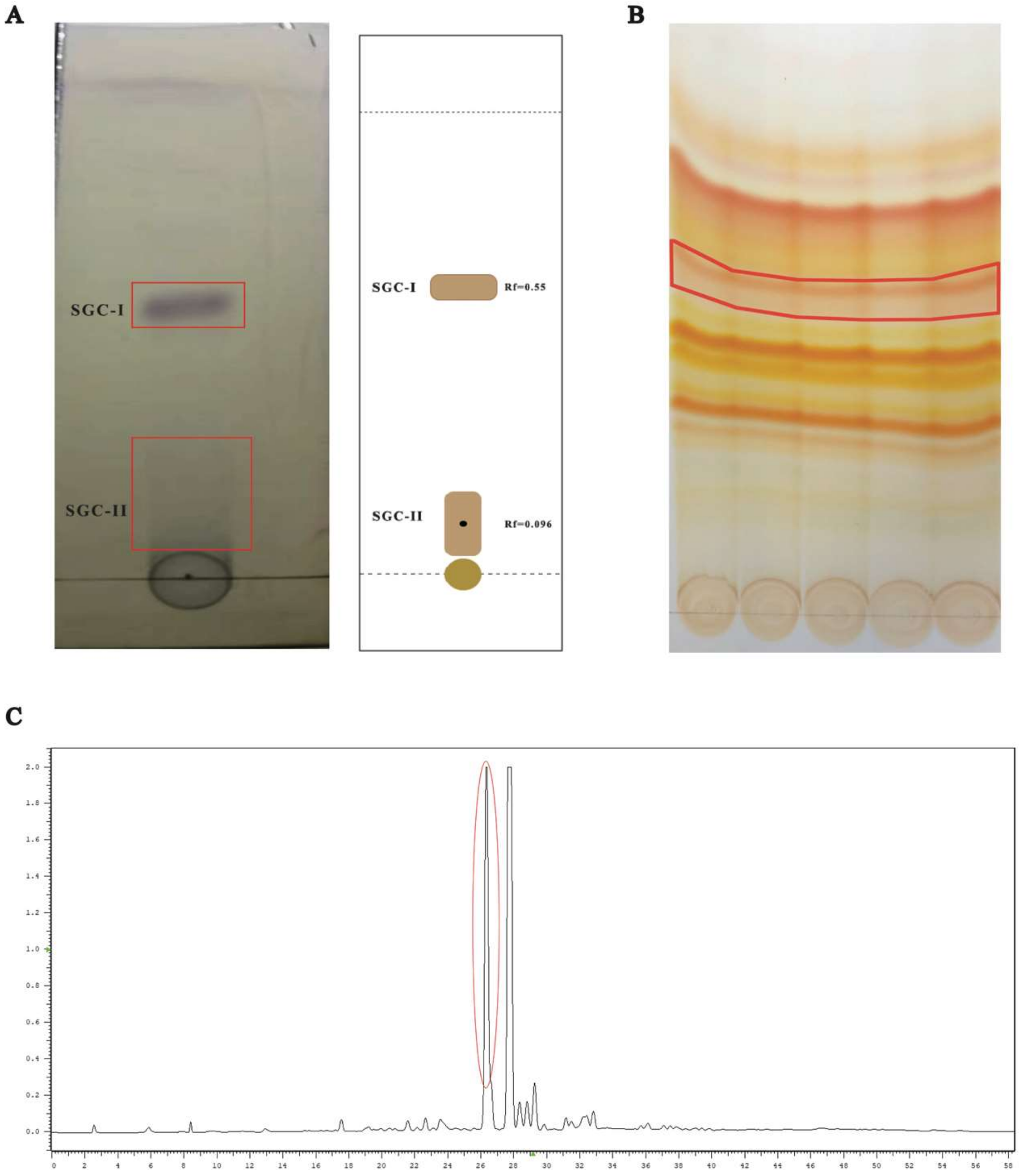

Figure S1. Isolation and activity verification of Halorotetin A. (**A**) The two parts of TE obtainted by silica gel column chromatography, the SGC-II was the active part. (**B**) The different parts of SGC-II obtained by PLC separation, and the PCL-VI was exhibited in the red circle. (**C)** The different parts of PLC-VI obtained by semipreparative-HPLC, and the Halorotetin A was exhibited in the red circle.

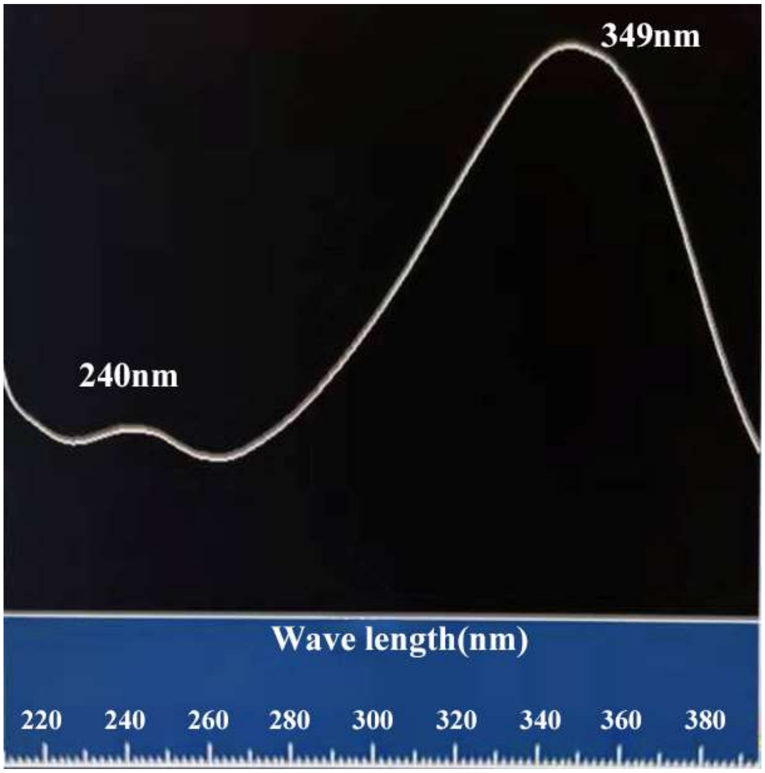

Figure S2. UV curve of Halorotetin A in Methanol

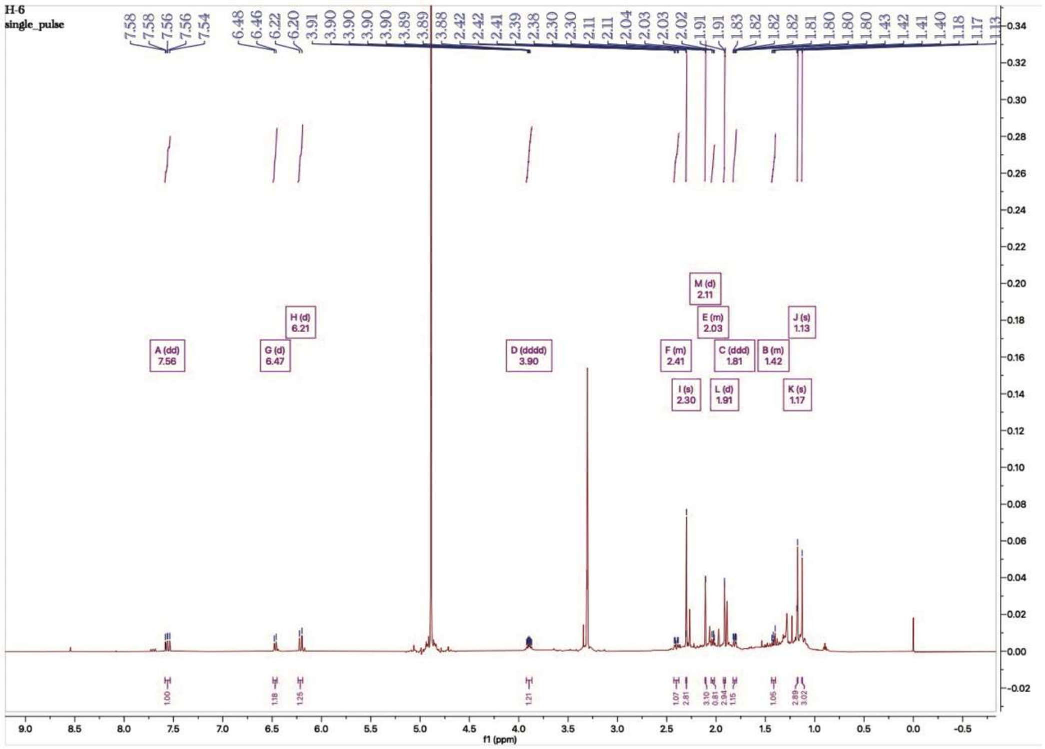

Figure S3. ^{1}H NMR spectrum of Halorotetin A in Methanol-d_4

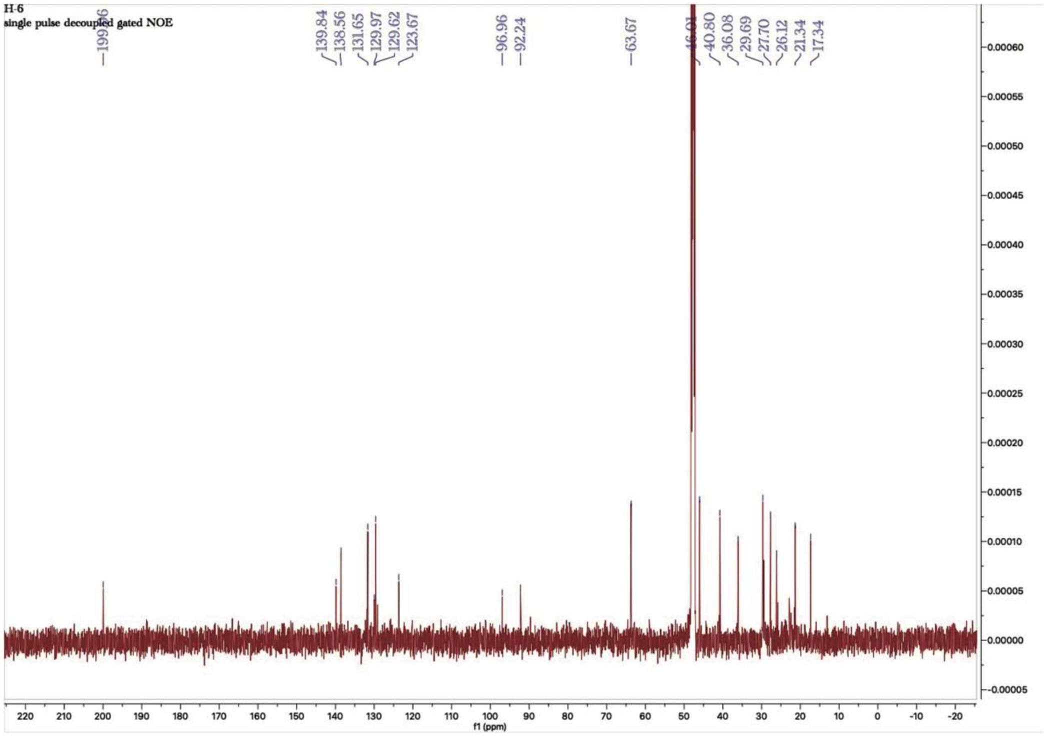

Figure S4. ^{13}C NMR spectrum of Halorotetin A in Methanol-d_4

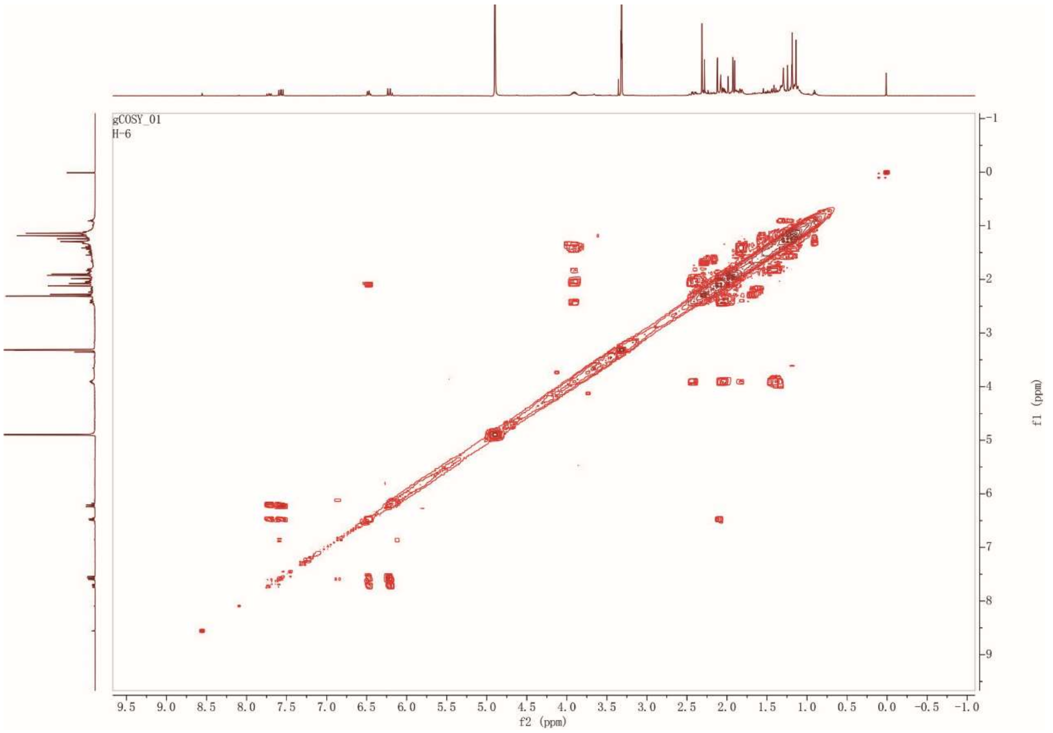

Figure S5. ^{1}H-^{1}H COSY spectrum of Halorotetin A in Methanol-d_4

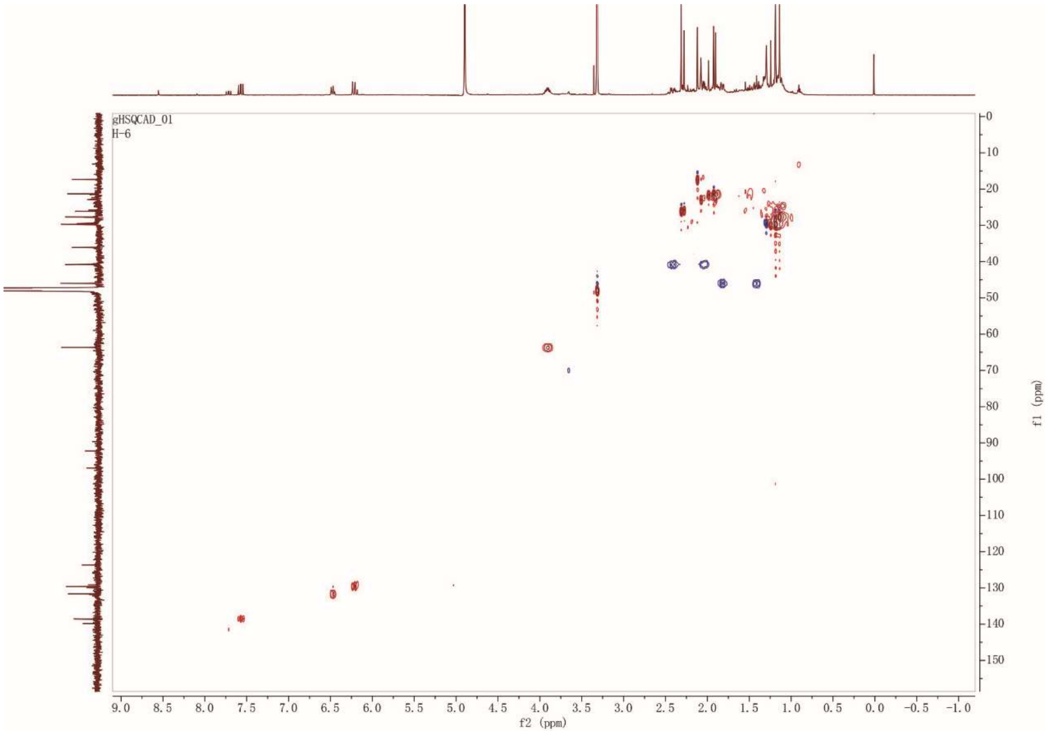

Figure S6. HSQC spectrum of Halorotetin A in Methanol-d_4

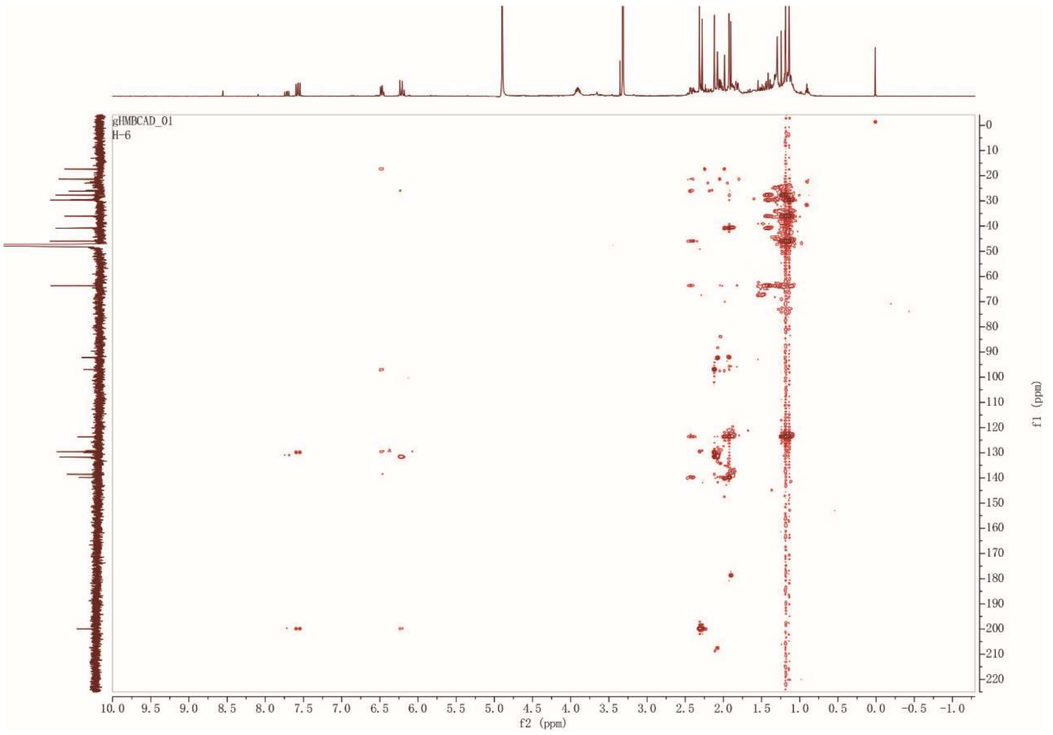

Figure S7. HMBC spectrum of Halorotetin A in Methanol-d_4

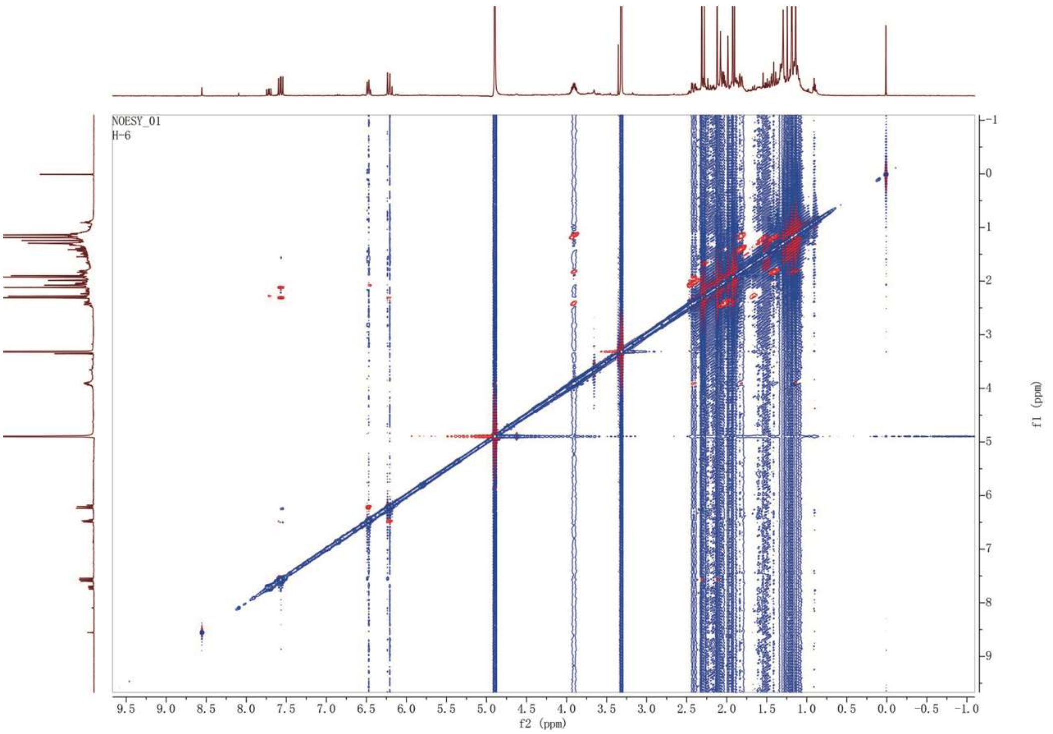

Figure S8. NOESY spectrum of Halorotetin A in Methanol-d_4

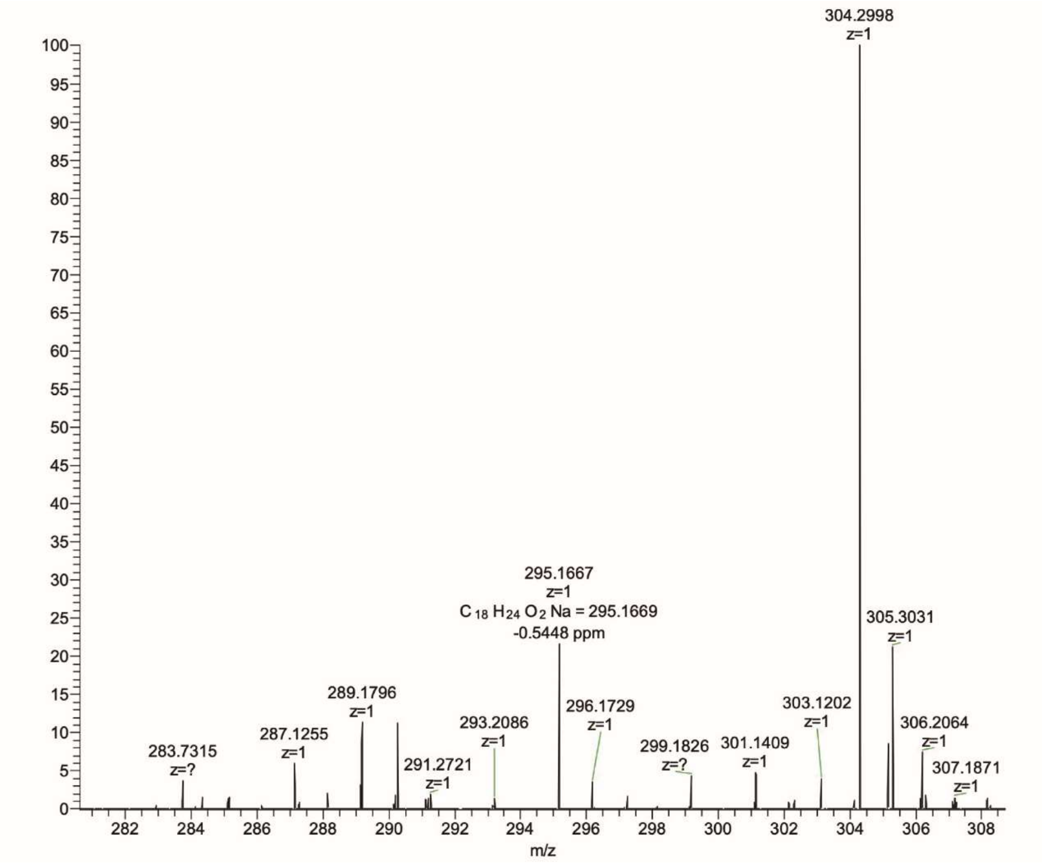

Figure S9. HRESIMS [M+Na]+ spectrum of Halorotetin A

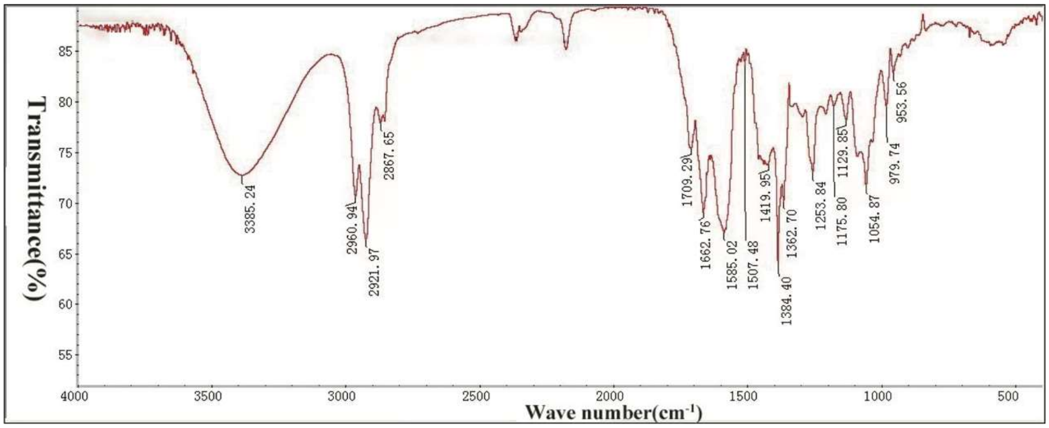

Figure S10. Infrared absorption (IR) spectrum of Halorotetin A

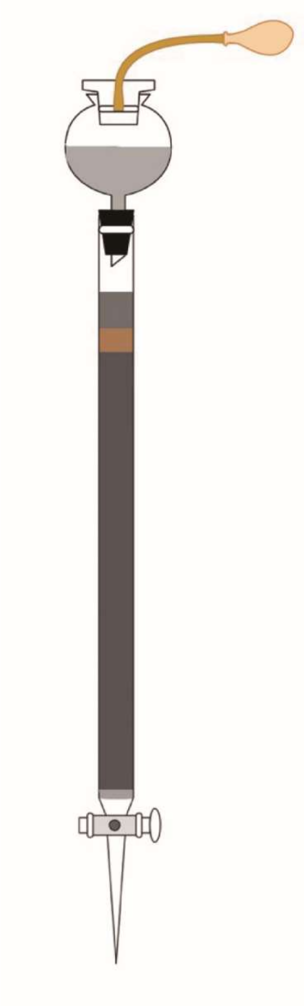

Figure S11. Schematic representation of the silica gel column (24mm×600mm). From top to bottom, the 60-80 mesh silica gel layer, the sample layer, and the 300-400 mesh silica gel layer, respectively.

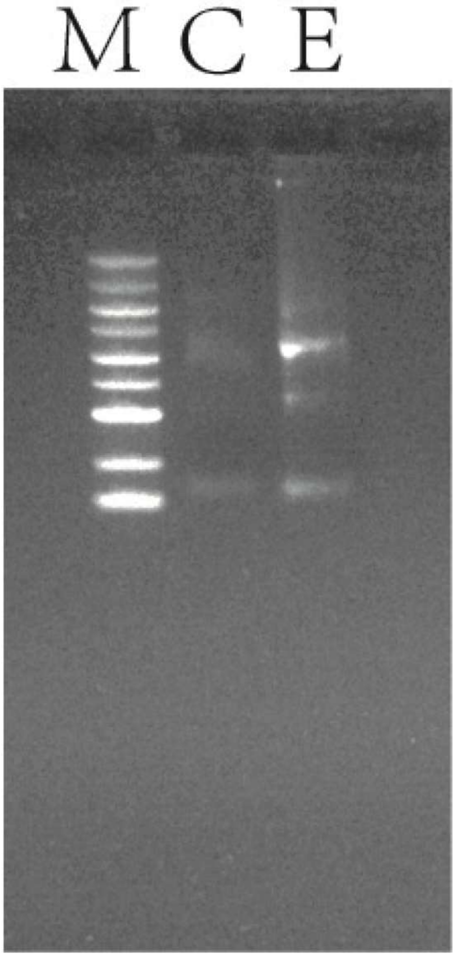

Figure S12. RNA electrophoresis pattern. The RNA was extracted from HepG-2 cells in the control group and the experimental group. RNA quality was determined by 1% agarose gel electrophoresis. M represents Maker, C represents RNA from the control group, and E represents RNA from the experimental group.

Table S1. The protocol of HPLC for Halorotetin A

Time (min)	A Pump （Acetonitrile）	B Pump（Water）	Flow Velocity
0.0	20	80	2ml/min
10	50	50	
25	70	30	
40	100	0	
55	100	0	
60	100	0	

Table S2. Primers used for qRT-PCR analysis

P1K3CA	Forward: TGTTACTCAAGAAGCAGAA<br>Reverse: GCCTACTGGTTCAATTACT
PTEN	Forward: ACGAACTGGTGTAATGATAT<br>Reverse: TGTCTCTGGTCCTTACTT
AXIN	Forward: CCTATCCCTCCTTCCTTA<br>Reverse: GGCAGGTATCCAGATATG
ARID1A	Forward: AAGGAGATTGGTGGATTG<br>Reverse: GACACTGGATATACTGCTT
C-Myc	Forward: AACAAGAAGATGAGGAAGAA<br>Reverse: AGAAGGTGATCCAGACTC
C-Met	Forward: TCAGCGATCTTCTCTACT<br>Reverse: CCTCATCATCAGCGTTAT
TP53	Forward: CGTGTGGAGTATTTGGAT<br>Reverse: ATGTAGTTGTAGTGGATGG
KEAP1	Forward: GACAAACCGCCTTAATTC<br>Reverse: CAGCAGCATAGATACAGT
ARID2	Forward: ATGTATCTCCTGCTCCTT<br>Reverse: CCACAGTATGAACACCTT
